# Cystic fibrosis in Tuscany: evolution of newborn screening strategies over time to the present

**DOI:** 10.1186/s13052-020-00948-8

**Published:** 2021-01-06

**Authors:** Matteo Botti, Vito Terlizzi, Michela Francalanci, Daniela Dolce, Maria Chiara Cavicchi, Anna Silvia Neri, Valeria Galici, Gianfranco Mergni, Lucia Zavataro, Claudia Centrone, Filippo Festini, Giovanni Taccetti

**Affiliations:** 1 Tuscany Support Cystic Fibrosis Service, Department of Pediatrics, Leghorn Hospital, Leghorn, Italy; 2 Tuscany Referral Cystic Fibrosis Center, Anna Meyer Children’s Hospital, Florence, Italy; 3 Diagnostic Genetics Unit, Careggi University Hospital, Florence, Italy; 4 Department of Pediatrics, Anna Meyer Children’s Hospital, University of Florence, Florence, Italy

**Keywords:** Cystic fibrosis, Newborn screening for cystic fibrosis CF NBS, CFTR allelic heterogeneity, Immunoreactive trypsinogen, DNA molecular analysis

## Abstract

**Background:**

Cystic fibrosis (CF) is a life-threatening disease affecting about 1:3000 newborns in Caucasian populations. The introduction of newborn screening for cystic fibrosis (CF NBS) has improved the clinical outcomes of individuals with CF through early diagnosis and early treatment. NBS strategies have been implemented over time. CF NBS was introduced extensively in 1984 in Tuscany, a region with 3.7 million people, characterized by a high allelic heterogeneity of CFTR gene.

**Aim and methods:**

The aim of the study is to present the results from 34 years (1984–2018) of CF NBS, retrospectively evaluating the sensitivity, specificity and predictive values of the tests. In particular, we studied the impact of the introduction of DNA molecular analysis in NBS in a region with high allelic heterogeneity, such as Tuscany.

**Results:**

Over these 34 years, 919,520 neonates were screened, using four different NBS strategies. From 1984 to 1991, CF NBS was performed by the determination of albumin on dried meconium (sensitivity 68.75%; specificity 99.82%). Subsequently, the analysis of immunoreactive trypsinogen on a blood spot was adopted as CF NBS protocol (sensitivity 83.33%; specificity 99.77%). From 1992 to 2010, this strategy was associated with lactase meconium dosage: *IRT1/IRT2 + LACT protocol* (sensitivity 87.50%; specificity 99.82%). From 2011, when the existing algorithm was integrated by analysis of CF causing variants of the CFTR gene (IRT1/IRT2 + LACT + IRT1/DNA protocol), a substantial improvement in sensitivity was seen (senisitivity 96.15%; specificity 99.75%). Other improved parameters with DNA analysis in the NBS programme, compared with the previous method, were the diagnosis time (52 days vs. 38 days) and the recall rate (0.58 to 0.38%).

**Conclusion:**

The inclusion of DNA analysis in the NBS was a fundamental step in improving sensitivity, even in a region with high allelic variability.

## Introduction

Cystic fibrosis (CF) is the most common life-threatening autosomal recessive disorder in the Caucasian population [1].

The classic CF phenotype is characterized by lung disease, exocrine pancreatic insufficiency associated with nutrient malabsorption contributing to malnutrition and impaired growth [2]. This genetic disease is caused by variants of the gene encoding for the cystic fibrosis transmembrane conductance regulator (*CFTR*) [3]. The most common *CFTR* pathogenetic variant associated with cystic fibrosis is p.Phe508del (F508del) [4]. In the 1970s, it was recognized that asymptomatic affected neonates could be detected at birth by neonatal screening [5]. The effect of newborn screening for cystic fibrosis (CF NBS) has been extensively studied and debated; the benefits have been unequivocally shown [6, 7].

Newborn screening strategies have been implemented since the early 1970s, initially using meconium albumin test and later immunoreactive trypsinogen in blood spot (IRT). Currently, the IRT test is usually performed in combination with a second retesting of IRT (IRT2), pancreatitis-associated protein on blood (PAP) or *CFTR* variants analysis (DNA) [8]. The screening algorithm can be integrated by a pancreatic meconium lactase assay (LACT), as described by *Pederzini* et al [9]. Once a positive CF NBS result has been found, sweat chloride testing must be performed to establish a CF diagnosis [10].

The IRT assay, the first step of CF NBS, has better sensitivity than screening test used previously [11]. A raised immunoreactive trypsinogen level in the first week of life (IRT1) is a sensitive, but not specific, test for CF. IRT values decrease in infants without CF over the first 4 weeks of life, but remain high in those with CF. A second evaluation of IRT (IRT2), after 3–4 weeks, avoids an inappropriate number of families coming to hospital for the sweat test [12, 13].

With the identification of the gene associated with CF (*CFTR*), *Gregg* et al. proposed using DNA testing in a new IRT/DNA 2-tier CF NBS algorithm [14]. Currently in North America, where CFTR variability is low (about 80% of F508del allele frequency in CF patients), DNA analysis in CF NBS is performed more frequently than in Europe [8].

Substantial expansion of screening for CF has been reported, with countries adopting different approaches depending on their target populations [7, 8]. Worldwide, the sensitivity of CF NBS varies between 86.6 and 97.5% depending primarily on differences in IRT cut-offs and the detection rate of genetic *CFTR* analysis panels [15, 16].

In Italy, the reported frequency of CF is 1:4176 live births [17]. Several screening protocols are used in different Italian regions. Tuscany is a region in central Italy with a population of 3,729,641 inhabitants (2018). There is a high variability of the *CFTR* variants in Tuscany: only about half of Tuscan patients have at least one allele with the F508del [18, 19]. CF NBS started in Tuscan in 1982, and since 1984, there has been a centralized screening programme in Florence for all Tuscan newborns. The primary aim of this study is to evaluate how the several CF NBS strategies in Tuscany performed from 1984 to 2018 and analyze the sensitivity, specificity and predictive values of each programmes. The introduction of DNA analysis in a region such as Tuscany with high allelic heterogeneity, was an interesting challenge [20].

## Aim and methods

We reviewed the complete NBS database of the CF Center of Florence from 1984 to 2018, and retrospectively evaluated the number of total newborn screenings, the true positives (TPs), the false positives (FPs), the true negatives (TNs), the false negatives (FNs) and the CF newborn with meconium ileus. Subsequently, we calculated the sensitivity (the capability of the test to identify affected subjects), specificity (the ability of a test to give a negative result in unaffected subjects), negative predictive values (NPV) and positive predictive value (PPV). The CF diagnosis was confirmed by the sweat test (*Gibson and Cook method*) [10] performed at the CF Center of Florence, where all patients resident in Tuscany with suspected CF were referred.

Overall, in Tuscany four CF NBS strategies were applied in the 34 years (from 1984 to 2018).

From 1984 to 1991, CF NBS was performed by the determination of albumin on dried meconium by semiquantitative radial immunodiffusion assay (SQRID). In 1991, the protocol (IRT1/IRT2) based on immunoreactive trypsin assay (IRT1) on dried blood spots began, which was followed, if positive, by a retesting of IRT (IRT2). The initial step of this algorithm was IRT1 measured from a blood spot sample taken on the third day of life. In 1992, this protocol was integrated with meconium lactase dosage (IRT1/IRT2 + LACT). The lactase test (LACT) is still performed in the CF NBS programme in some regions in Italy, as for example northeastern Italy [9].

Our laboratory, as in others worldwide, had advocated the continuous monitoring of IRT1 values and altering the cutoff accordingly to achieve a predetermined percentage of infants referred. The IRT1 cut-off of was set at the 99.0th centile, changing every 4–6 months according to NBS results and on the basis of the seasons, as described in literature [21, 22]. In 1992–1993 we used a colorimetric method for dried blood spot analysis of IRT1 (*Medical System*, IRT1 99th: 55 ng/mL). Successively, a fluorimetric method was used for IRT1 analysis (*Delfia* in 1994–2005, IRT1 range 99th: 54–62 ng/mL; *Auto-Delfia* in 2006–2013, IRT1 range 99th: 57–63 ng/mL; *GSP instrument-Perkin Elmer* 2014–2018, IRT1 range 99th: 49–50 ng/mL). The IRT2, taken at three or 4 week of age, followed the same procedure as the initial IRT1 assay (1992–2013 cut-off 29 ng/mL; 2014–2018 cut-off 23 ng/mL). The meconium of all children was collected, but meconium lactase activity was evaluated only in those with an IRT1 value >99th percentile. Meconium lactase was detected by glucose production after incubation of meconium with lactose (cut off 0.5 U/g), as described by *Pederzini* et al. [9].

**Fig. 1:**
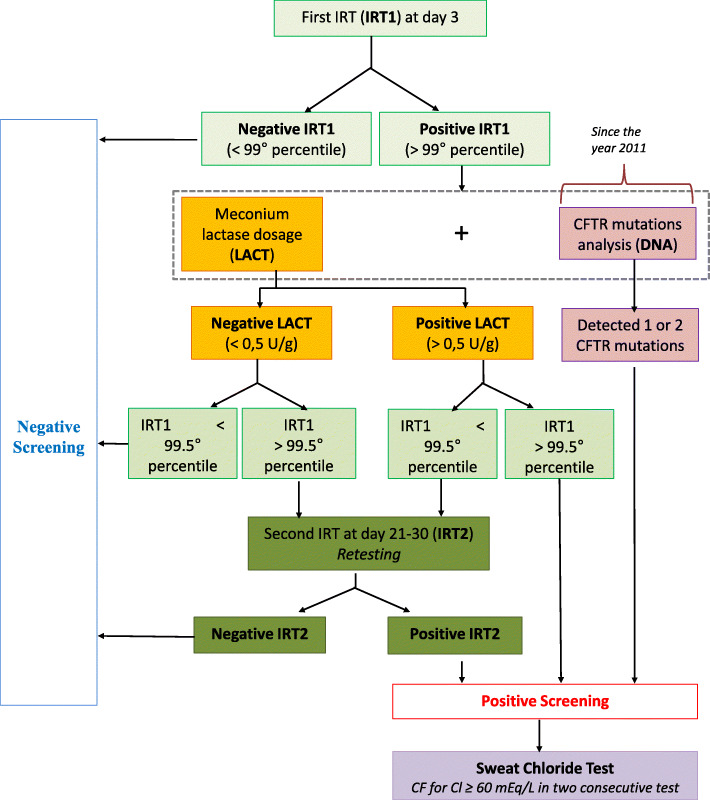
CF NBS algorithm adopted in Tuscany from 2011

In 2011, a pilot project was initiated to assess the diagnostic impact of DNA analysis on the CF NBS algorithm. In these 8 years (2011–2018), we had two “parallel” protocols simultaneously active: *protocol 1* IRT1/IRT2 + LACT and *protocol 2* IRT1/DNA. This algorithm (IRT1/IRT2 + LACT + IRT1/DNA) is described in detail in Fig. 1. In cases where the IRT value was >99th centile, we screened for a panel of *CFTR* pathogenetic variants with a detection rate of 80% until 2015 (38 CF causing variants), 88% from 2016 to 2018 May (66 CF causing variants), and 90% until December 2018 (272 CF causing variants). Dependent on whether *CFTR* variants were found on one or both alleles, referral to the CF Center for treatment, sweat testing, and possibly further genotyping was recommended.

The TPs included all CFSPID (cystic fibrosis screening positive inconclusive diagnosis) that evolved in CF during the follow-up (until December 31st, 2019) [19, 23].

The FNs were all the patients, born in Tuscany over the 34 years, who screened negative and had a CF diagnosis confirmed between 1984 and December 31st 2019 by a sweat chloride concentration ≥ 60 mmol/L and/or 2 *CFTR* pathogenetic variants in trans [24]. With these time intervals of the study, we allowed for any hypothetical FNs born in 2018 to emerge in the successive year (by the 31st December 2019). The occurrence of FN results in CF newborns with meconium ileus is reported in the literature [25], however, it is not a concern as these patients must always be investigated with the sweat test [7]. Therefore, patients with CF and meconium ileus at birth were considered separately.

A more detailed analysis of the screening algorithm and the data available has been undertaken since 2011, the official year of introduction of the DNA analysis in the CF NBS strategy. We evaluated the number of IRT retesting (IRT2) during these years. We also assessed retrospectively, from 2000 to 2018, the time (in days) between the birth and the CF diagnosis of TPs. The CFSPID were excluded from this analysis. Statistical analyses with ANOVA tests were used for comparison of these data. A *p*-value of less than 0.05 was considered statistically significant.

## Results

In Tuscany, 919,520 CF NBS were performed over 34 years. CF was diagnosed in 258 patients, where 199 (77.1%) screened positive in the neonatal programme, 28 (10.9%) presented with meconium ileus and there were 31 (12.0%) FNs.

Tuscany CF NBS with SQRID protocol had 68.75% sensitivity, 99.82% specificity and 10.09% PPV (Table 1, second column). The IRT1/IRT2 protocol showed 83.33% sensitivity, 99.77 specificity and PPV 10.27% (Table 1, third column); this protocol in 1992 was integrated with meconium lactase dosage (IRT1/IRT2 + LACT) and the data showed 87.50% sensitivity, 99.78% specificity and PPV 9.16% (Table 1, fourth column). From 2011, the existing algorithm was integrated with analysis of CF-causing variants of the CFTR gene and the CF NBS reached a 96.15% sensitivity, 99.75% specificity and PPV 8.09% (Table 1, fifth column). The comparison of overall results (sensitivity, specificity, NPV, PPV) of these four strategies of CF NBS, relating to the period 1984–2018, are summarized in Table 1.

**Table 1:** Results of the various screening strategies of CF NBS adopted over time (1984–2018) by the CF Referral Tuscan Center of Florence. () percentage related to all CF cases of the period in column

	SQRID (1984–1991)	IRT1/IRT2 (1991–1994)	IRT1/IRT2 + LACT (1992–2010)	IRT1/IRT2 + LACT + IRT1/DNA (2011–2018)	Total
Newborns screened for CF	113,288	59,546	514,841	231,845	919,520
Total CF cases	37	19	143	59	258
Newborns with negative screening	113,070	59,400	513,619	231,227	917,316
Non CF newborns with negative screening (**TNs**)	113,060	59,397	513,603	231,225	917,285
Number of *retesting* (IRT2)	–	936	2977	906	4819
Newborns with positive screening (number of sweat test)	218	146	1222	618	2204
Non CF newborns with positive screening **(FPs)**	196	131	1110	568	2005
CF newborns with positive screening (**TPs**)	22 (59.4%)	15 (79.0%)	112 (78.3%)	50 (84.7%)	199 (77.1%)
CF newborns with negative screening (**FNs**)	10 (27.0%)	3 (15.8%)	16 (11.2%)	2 (3.4%)	31 (12.0%)
CF newborns diagnosed for meconium ileus	5 (13.5%)	1 (5.2%)	15 (10.5%)	7 (11.9%)	28 (10.9%)
**Sensitivity (%)**	68.75	83.33	87.50	96.15	–
**Specificity (%)**	99.82	99.77	99.78	99.75	–
**Positive predictive value (%)**	10.09	10.27	9.16	8.09	–
**Negative predictive value (%)**	99.99	99.99	99.99	99.99	–

**Fig. 2:**
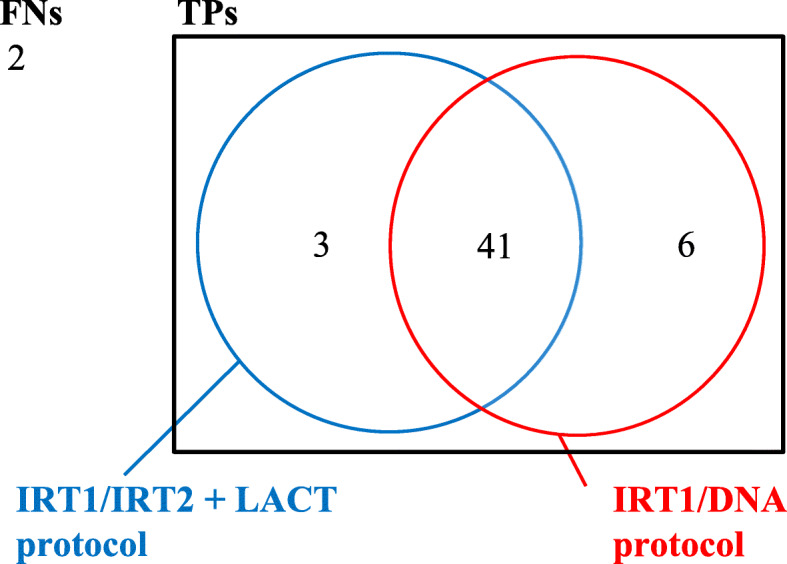
Distribution of CF diagnoses (TPs and FNs) in the CF NBS protocol, in the 2011–2018 period with coexistent *IRT1/IRT2 + LACT protocol* and *IRT1/DNA protocol*

By separately analyzing the data of the period 2011–2018 of both “*parallel*” protocols (IRT1/IRT2 + LACT and IRT1/DNA), we can independently evaluate the contribution of DNA molecular analysis on screening. These results are reported in Table 2. Figure 2 schematically shows the distribution of TPs and FNs in relation to the screening algorithms in the 2011–2018 period.

**Table 2:** Data of the 2011–2018 CF NBS algorithm: *IRT1/IRT2 + LACT protocol* and *IRT1/DNA protocol* separately

	CF NBS 2011–2018		
	IRT1/IRT2 + LACT	IRT1/DNA	IRT1/IRT2 + LACT + IRT1/DNA (actual strategy)
Newborns screened for CF	231,845	231,845	231,845
Newborns with negative screening	231,370	231,604	231,227
Non CF newborns with negative screening (**TNs**)	231,362	231,599	231,225
Number of *retesting* (IRT2)	906	–	906
Number of sweat tests (newborns with positive screening)	475	241	618
Number of sweat tests after positive IRT1 + LACT	157	–	–
Number of sweat tests after positive *retesting* (IRT2)	318	–	–
Non CF newborns with positive screening **(FPs)**	430	194	568
CF newborns with positive screening (**TPs**)	44	47	50
CF newborns with negative screening (**FNs**)	8	5	2
CF newborns diagnosed for meconium ileus	7	7	7
**Sensitivity (%)**	84.61^a^	90.38^a^	96.15
**Specificity (%)**	99.81^a^	99.91^a^	99.75
**Positive predictive value (%)**	9.26^a^	19.50^a^	8.09
**Negative predictive value (%)**	99.99^a^	99.99^a^	99.99

^a^These values are different from those of Table 2 of *Terlizzi* et al (2019) [22] because our analysis covered 1 year longer (2011–2018 vs. 2011–2017)

The impact of DNA analysis on the prevalence of CFSPID infants in Tuscany from 2011 to 2017 has already been studied [19]. Clinical and genotypical features of FNs, born in Tuscany from 1992 to 2018, have already been discussed in *Taccetti* et al [26].

It was found that from 2000 to 2010 (IRT1/IRT2 + LACT protocol), the diagnosis was given to parents on average at 51.9 days of life (with a minimum of 28 days), while in the period 2011–2017 (protocol with DNA), it was given at 37.9 days (with a minimum of 18 days). This difference was statistically significant by ANOVA test (*p* < 0.00017).

## Discussion

The overall CF incidence in this period was 1:3564 newborns, which is higher than the Italian national average shown in the latest report of Italian CF Registry (1:4176) [17]. Large-scale CF NBS, extended from the late 1980s to all Tuscan births, allowed early diagnosis of CF in most cases. In fact, only 31 (12%) out of 258 patients born in the period 1984–2018 were FNs at screening. The early diagnosis of CF in all the other cases (77.2% with positive CF NBS and 10.8% with meconium ileus in the first days of life) allowed the CF Regional Center to start specific CF treatments (nutritional, physiotherapy, saline supplementation, microbiological surveillance).

Across Europe, there has been an increase in public CF NBS programmes, with variable strategies and outcomes that reflect the different approaches [27]. Our results show that in Tuscany, screening strategies have gradually improved. Indeed, the percentage of FNs over the years has gradually decreased as NBS sensitivity has gradually increased. We underline the importance for NBS laboratories to receive a feedback on new CF cases not diagnosed with screening, which allow the quality of the NBS programmes to be evaluated.

In the IRT1/IRT2 strategy, the inclusion of lactase assay on meconium (LACT) reduced the occurrence of false-positive test results, as already described in literature [9, 11].

DNA analysis is performed on the same blood spot as IRT1, thus the increasing sensitivity and specificity, detection of *CFTR* pathogenetic variants at birth, and improving timeliness of diagnosis [15, 28]. Protocols that use DNA analysis also detect carriers, which is considered an unwanted effect for NBS programmes [8]. NBS strategies with DNA analysis have high economic costs compared with others, but all screening strategies are cost-effective when compared to the non-screening option [29]. The increased implementation of CF NBS has led to the identification of infants with a positive NBS test but inconclusive diagnostic testing (CFSPID) [23].

From 2011, the year in which DNA molecular analysis was introduced in Tuscany, only 2 cases of CF were FNs in 59 total cases of NBS. Therefore, the sensitivity of the screening with the last protocol adopted (2011–2018) is 96.15% (compared to 87.50% of the previous period). This last value is in line with the current recommendations of *ECFS* (recommended sensitivity > 95%) [7]. It should be noted that this high sensitivity, referring to the period 2011–2018, could be reduced in the coming years with the possible diagnosis in the future of new cases of CF with negative screening, which were still asymptomatic on the 31st December 2019. The choice to consider newborn with meconium ileus separately, independent of the NBS results, may have positively influenced the value of sensitivity. The specificity of the CF NBS has always been very high over the years (> 99.7%), with each screening test algorithm used.

The ECFS established that NBS programmes should aim for a minimum positive predictive value of 0.3 and the timeliness of diagnosis should be no later than 58 days after birth, or even better, 35 days [7]. Analyzing the results of the study, we see that the recall rate (infants recalled to perform the retesting, IRT2) was excessively high (1.6%) in the IRT1/IRT2 period, but subsequently decreased to 0.58% (1992–2010 period) and 0.39% (2011–2018 period). A low recall rate, as occurred in Tuscany with the latest screening strategy, is to be considered very positive, as it reduces the number of infants undergoing retesting, an event that causes anxiety in the family [30].

The two “*parallel*” protocols in 2011–2018 worked synergistically at reducing FNs. As shown in Fig. 2, IRT1/DNA allowed the diagnosis of six more CF patients, who would have been missed with only the IRT/IRT2 + LACT algorithm, while with IRT1/IRT2 + LACT, three further infants with CF were identified (who would have been missed with only IRT1/DNA).

From these data, it can be deduced that the introduction of DNA analysis had a substantial effect on improving the sensitivity of NBS in Tuscany. At the same time, it did not improve the PPV (the probability that an infant with a positive screening result is actually affected), which was 8.09%. The main reason for the low PPV value is that the current Tuscan screening algorithm is still a “*parallel double protocol*” pilot project, therefore the subjects who have to perform the sweat test are inevitably numerically high. It should be noted (Table 2) that if there had been only the IRT1/DNA protocol, as in many States [27], the PPV would have been 19.5%, with a sensitivity of 90.38% (which is, however, higher compared to the past).

In Italy, the importance of having *CFTR* variants analysis in NBS lies mainly in the extreme allelic variability of the *CFTR* gene, compared to other regions such as those of Northern Europe, the United States and Canada, where the percentage of CF carriers with at least one F508del is about 70–80%. This extreme genetic variability is even more evident in some regions, such as Tuscany [18, 19, 31], making the application of screening programmes with DNA analysis more difficult and complex. For this reason, to allow such a considerable increase in sensitivity, it was necessary to analyze a larger number of *CFTR* pathogenetic variants, which gradually increased in number over the years (272 causing variants from May 2018). An example is the S737F variant, common in Tuscany [31], recently introduced in the NBS panel, which is associated with symptoms of hypochloremic alkalosis in the first years of life, mild CF phenotype in teenage years and a residual function of *CFTR* protein.

We also analyzed the five FNs of IRT1/DNA subjects, two of them, both born before May 2018 (month in which the detection rate increased to 90%), could have been identified today because their variants (Q39X and 1898 + 1G- > A) have been inserted in the current panel of 272 CF causing variants.

The CF NBS performed in the 2011–2018 period highlighted, among the 568 FPs, 194 carrier infants identified by IRT1/DNA, carrying one pathogenetic variant of the *CFTR* gene (heterozygous, not affected by CF). A potential advantage was that the parents of these infants were able to access genetic medical counseling so that they had the implications of the child’s carrier status explained to them [32]. Particular attention had to be paid to underlining the differences between carrier and CF affected patients so as not to create the parents unnecessary anxiety. Attention also had to paid to possible planning of a future pregnancy, in particular by analyzing the carrier status of both parents. In our study, we did not analyze this aspect of prenatal and postnatal genetic counseling within the CF NBS programmes.

An effect of the introduction of the DNA analysis was also the age of at diagnosis, as described in literature [8]. Since 2011, CF patients were diagnosed by NBS on average 14 days earlier (38 days vs. 52 days), compared to the period 2000–2010 (a difference that is statistically significant, *p* < 0.05). This reduction in the time of diagnosis could be an advantage in countries with hot climates, such as Tuscany, to prevent salt loss syndrome through earlier salt supplementation.

## Conclusion

NBS is a fundamental public health instrument for the early diagnosis of CF. In Tuscany a radical innovation of the protocol occurred in 2011, when the classic IRT1/IRT2 + LACT algorithm was integrated with the analysis of pathogenetic variants of the *CFTR* gene (IRT1/DNA). This change led to many substantial improvements: FNs decreased significantly compared to the previous period and therefore sensitivity (87.50% vs. 96.15%) increased. Other improved parameters were the time for diagnosis (52 days vs. 38 days) and the recall rate (0.58 to 0.38%). There was no improvement in the PPV with IRT1/IRT2 + LACT + IRT1/DNA (9.16% vs. 8.09%). DNA analysis has made it possible to identify 194 carrier infants, and therefore consequently to carry out genetic counseling for the family in anticipation of future pregnancies. The inclusion of DNA analysis in the NBS was a fundamental step in improving sensitivity even in a region with high allelic variability, such as Tuscany.

## Data Availability

Newborn screening database of the Cystic Fibrosis Center of Florence; this database (computer and paper Archive) is not available online, it is available only by specific request.

## Abbreviations

CF: Cystic fibrosis
NBS: Newborn screening
*CFTR*: Cystic fibrosis transmembrane conductance regulator
SQRID: Semiquantitative radial immunodiffusion assay
IRT: Immunoreactive trypsinogen
LACT: Pancreatic meconium lactase assay
TPs: True positive cases
FPs: False positive cases
TNs: True negatives
FNs: False negatives
NPV: Negative predictive values
PPV: Positive predictive value
SPID: Screening positive inconclusive diagnosis
